# Peroxisome proliferator‐activated receptors as therapeutic target for cancer

**DOI:** 10.1111/jcmm.17931

**Published:** 2023-09-12

**Authors:** Yuqing Wang, Feifei Lei, Yiyun Lin, Yuru Han, Lei Yang, Huabing Tan

**Affiliations:** ^1^ Department of Internal Medicine Montefiore Medical Center, Wakefield Campus Bronx New York USA; ^2^ Department of Infectious Disease, Lab of Liver Disease, Renmin Hospital Hubei University of Medicine Shiyan China; ^3^ Department of Biomedical Sciences University of Texas, MD Anderson Cancer Center Houston Texas USA; ^4^ Qinghai Provincial People's Hospital Xining China

**Keywords:** cancer, combinational therapy, PPARs, therapeutic targets, tumor microenvironment

## Abstract

Peroxisome proliferator‐activated receptors (PPARs) are transcription factors belonging to the nuclear receptor family. There are three subtypes of PPARs, including PPAR‐α, PPAR‐β/δ and PPAR‐γ. They are expressed in different tissues and act by regulating the expression of target genes in the form of binding to ligands. Various subtypes of PPAR have been shown to have significant roles in a wide range of biological processes including lipid metabolism, body energy homeostasis, cell proliferation and differentiation, bone formation, tissue repair and remodelling. Recent studies have found that PPARs are closely related to tumours. They are involved in cancer cell growth, angiogenesis and tumour immune response, and are essential components in tumour progression and metastasis. As such, they have become a target for cancer therapy research. In this review, we discussed the current state of knowledge on the involvement of PPARs in cancer, including their role in tumourigenesis, the impact of PPARs in tumour microenvironment and the potential of using PPARs combinational therapy to treat cancer by targeting essential signal pathways, or as adjuvants to boost the effects of current chemo and immunotherapies. Our review highlights the complexity of PPARs in cancer and the need for a better understanding of the mechanism in order to design effective cancer therapies.

## 1 INTRODUCTION: PPAR ISOFORMS IN HEALTH AND DISEASES

**FIGURE 1:**
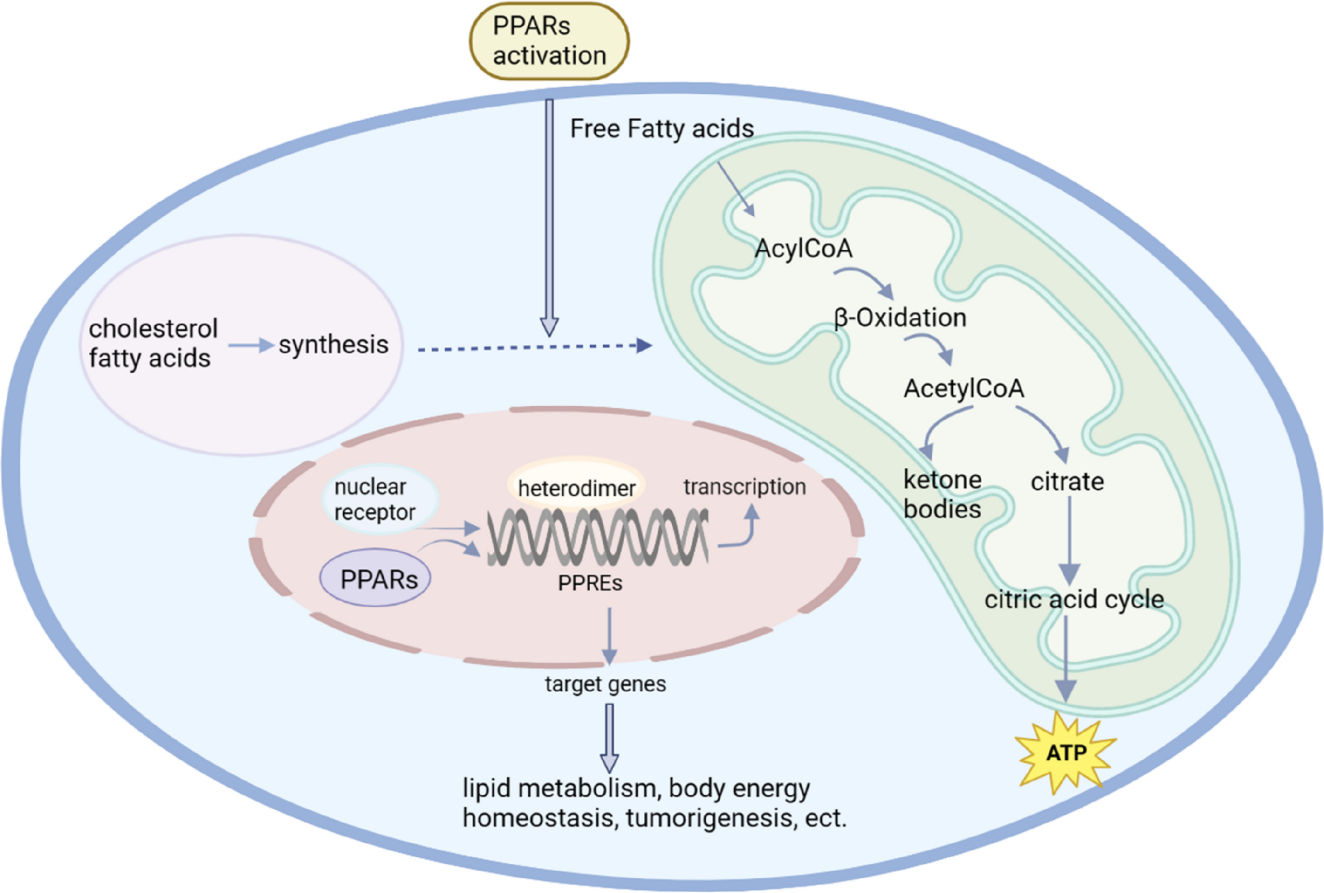
Activation of peroxisome proliferator‐activated receptors (PPARs). Upon activation, PPARs form heterodimers with nuclear receptors by binding to their ligands. The heterodimers then bind to peroxisome proliferator response elements (PPREs) in the promoter region, which regulates the expression of downstream target genes. These genes play crucial roles in various biological processes, such as lipid metabolism, body energy homeostasis and tumourigenesis. Figure was generated with BioRender.

Peroxisome proliferator‐activated receptors (PPARs) are transcription factors that belong to the nuclear receptor family, they can be activated by endogenous unsaturated and saturated fatty acids or synthetic ligands.^1^, ^2^, ^3^, ^4^ PPAR‐α, the first isoform of PPAR, was successfully cloned from the mouse liver in 1990 and is a new nuclear receptor that plays a key role in triglyceride and cholesterol homeostasis.^2^, ^5^ Two years later, all three PPAR isoforms, namely PPAR‐α, PPAR‐β/δ and PPAR‐γ, were isolated from the ovary and liver of Xenopus laevis.^5^, ^6^ Since then, 30 years of in‐depth research on PPAR has gradually unveiled its mystery. The expression of each PPAR isoform was found to be tissue specific. PPAR‐α is mainly expressed in liver, kidney and tissues involved in lipid oxidation. PPAR‐γ are found in macrophages, adipose tissue, vascular smooth muscle and tumours of various organ origins. Different from PPAR‐α and PPAR‐γ, the expression of PPAR‐β/δ has been reported in skeletal muscle, adipose tissue, heart, etc.^4^, ^7^ After ligand binding, PPARs form a heterodimer with nuclear receptor and regulate target gene expression by binding to specific consensus DNA sequences in the promoter, namely peroxisome proliferator response elements (PPREs) (Figure 1).^4^, ^8^ The function of PPARs is primarily accomplished through ligand binding. In the repressed state, the heterodimer binds to corepressor proteins, forming multi‐protein complexes containing histone deacetylase activity, which ultimately represses target gene transcription. Upon ligand binding, the heterodimer undergoes a conformational change, releasing the corepressor and enhancing the coactivator binding.^9^ Additionally, various post‐translational modifications (PTMs) also regulate the functions of PPARs. These modifications include phosphorylation, SUMOylation, ubiquitination, acetylation and O‐GlcNAcylation, which are found at multiple modification sites. The addition of these PTMs can have a wide spectrum of consequences on protein stability, transactivation function and co‐factor interaction. Furthermore, specific PTMs in PPAR proteins have complex roles in cancer and metabolism, as described in detail in this review.^10^

Currently, it has been established that PPAR isoforms are important in metabolism and body energy homeostasis.^11^ PPAR‐α regulates genes involved in fatty acid uptake, β‐oxidation and ω‐oxidation. It not only downregulates apolipoprotein C‐III, which regulates lipoprotein lipase hydrolysis, but also regulates genes involved in reverse cholesterol transport, such as apolipoprotein A‐I and apolipoprotein A‐II.^12^ PPAR‐δ activation can regulate HDL cholesterol levels and affect glycaemic control.^13^ Activation of PPAR‐δ significantly improves glucose tolerance and insulin resistance.^14^ PPAR‐γ is a major regulator of adipocyte differentiation, but recent studies have shown that its activation is also associated with the expression of many important genes that affect energy metabolism, including TNF‐α, leptin and adiponectin.^15^ PPAR‐γ can also induce cell cycle arrest by inhibiting cyclin‐dependent kinase activity in several tumour cell lines.^16^

PPAR agonists have been used in clinical practice for various purposes. For example, PPAR‐α agonists, such as fibrates, are used clinically to lower lipids and prevent atherosclerosis and cardiovascular disease,^11^, ^17^ whereas PPAR‐γ agonists, such as thiazolidinediones, reduce blood glucose levels mainly in skeletal muscle and adipose tissue by increasing insulin sensitivity.^18^ In addition, recent studies have found that PPAR isoforms are also crucial in a broad spectrum of biological processes, including cell proliferation and differentiation, signalling pathways involving fatty acid and eicosanoid, bone formation, tissue repair and remodelling.^19^ Therefore, the PPAR agonists have gained considerable interest as potential therapeutic candidates for neurodegenerative diseases,^20^ psychiatric disorders such as addiction and depression,^21^, ^22^, ^23^ liver^24^, ^25^ and kidney diseases^26^, ^27^ and autoimmune and inflammatory diseases.^28^, ^29^, ^30^ Furthermore, PPARs are closely related to cancer. Increasing evidence indicates that PPARs are involved in cancer cell growth, angiogenesis and tumour immune response and are essential in tumour progression and metastasis. In the scope of this review, we explored the effects and possible mechanisms of PPAR agonists in tumourigenesis and the tumour microenvironment (TME), we also analysed the latest evidence on the co‐administration of PPAR agonists with chemo, immune or other therapies and conducted a critical assessment of the existing knowledge gaps and progress in this area.

## 2 ROLES OF PPARs IN TUMOURIGENESIS

**FIGURE 2:**
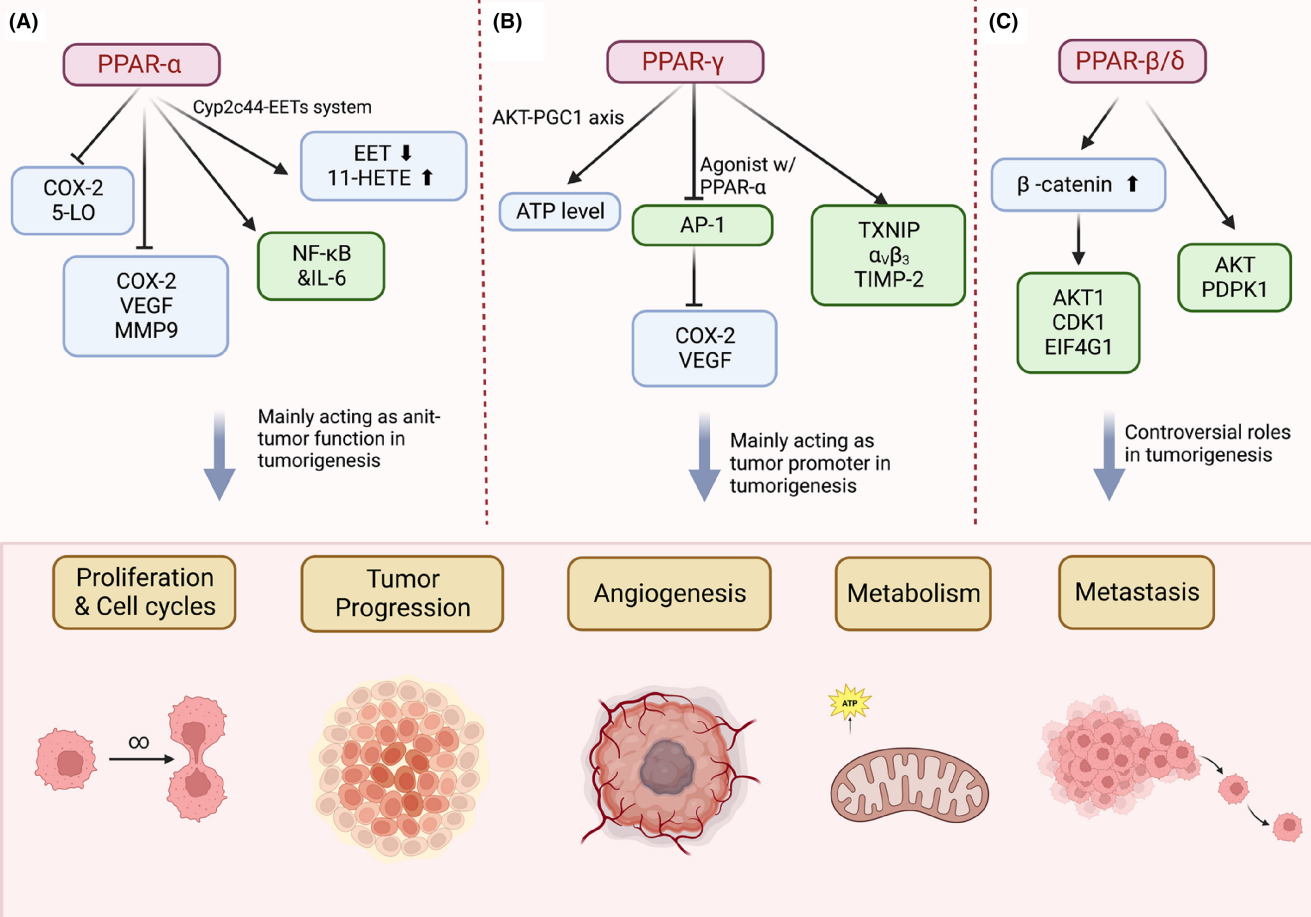
Roles of peroxisome proliferator‐activated receptors (PPARs) in tumourigenesis. Act as nuclear receptor, PPARs regulate lipid metabolism and are involved in tumour progression. (A) The isoform PPAR‐α regulates nuclear receptor NF‐κB and COX‐2‐related metabolism progression. PPAR‐α mainly plays an antitumour role during tumour progression. (B) The isoform PPAR‐γ increases ATP level through AKT involved signalling and reduces the expression of COX‐2/vascular endothelial growth factor (VEGF) by inhibiting AP‐1. PPAR‐γ mainly acts as a tumour promoter during tumour progression. (C) The isoform PPAR‐β/δ affects AKT mediated signalling and plays controversial roles in tumourigenesis. Figure was generated with BioRender.

Tumourigenesis is the process by which normal cells undergo a transformation and gain the malignant properties of proliferation, differentiation and metastasis.^31^ PPARs have been recognized as potential cancer therapies due to their key roles in metabolism and proliferation. Notably, different isoforms of PPARs play distinct roles in tumour progression across various cancer types. For example, the role of PPAR‐β/δ is controversial in multiple studies under different condition‐based disease types and research models. PPARs are widely involved in abnormal metabolism progress and could potentially act as a therapy option in various cancer types through versatile strategies. Here, we mainly focus on the roles of PPARs in cancer, specifically on cancer proliferation, metabolism and metastasis (Figure 2).

### 2.1 PPARs involve tumourigenesis through metabolism reprogramming

The dysregulated metabolism has been recognized as the hallmark of cancer^32^ and it has been shown that the fatty acid biosynthetic pathway is involved in an early stage of tumour progression.^33^ As nuclear receptors, PPARs involve in metabolic progress, which regulate the energy level and cell fate. Based on the different isoform and cancer type, PPARs exert a dual function in tumour progression, and the function of PPARs are determined by the various cancer types and research models. For example, a study using the orthograft prostate cancer model demonstrated that PPAR‐γ activation could stimulate the AKT‐PGC1 axis, resulting in increased ATP levels and enhanced mitochondrial biogenesis activities. The elevated ATP levels create an energetically favourable environment for tumour growth and metastasis.^34^ Moreover, in combination therapy, the co‐administration of PPAR‐α and PPAR‐γ agonists was found to inhibit activator protein‐1 (AP‐1), leading to reduced expression of cyclooxygenase‐2 (COX‐2) and vascular endothelial growth factor (VEGF). This combination of agonists was shown to reduce angiogenesis and induce apoptosis in a mouse model of OVCAR‐3 ovarian tumours. The activation of PPAR‐α and PPAR‐γ could also suppress the progression of solid ovarian tumour.^35^ In addition, PARA‐γ could support the cancer growth through the metabolism reprogramming of cancer‐associated fibroblasts (CAFs) and adipocytes.^5^

PPARs regulate multiple metabolic pathways which may further impact the cellular proliferation and promote metastasis in cancer progress. For example, activated PPAR‐α is deeply involved in the regulation of lipid metabolism in many healthy organs.^36^ Under the context of cancer, PPAR‐α regulates proliferation and cell cycles of tumour cells through inhibition of the prostaglandin biosynthesis and arachidonic acid metabolic pathway‐related enzyme COX‐2 and 5‐lipoxygenase (5‐LO).^37^ Similarly, in non‐small cell lung cancer, the activation of PPARα ligand reduced the production of proangiogenic epoxyeicosatrienoic acids (EET) and increased the hydroxyl 11‐hydroxyeicosatetraenoic acids (11‐HETE) through Cyp2c44‐EETs system, thus inhibiting tumour progression and metastasis (Figure 2A).^38^ More details on how PPARs involve tumourigenesis on these two aspects are as below.

### 2.2 Effects of PPARs in tumourigenesis involve tumour cell proliferation and survival

The abnormal proliferation is another hallmark of cancer. Cellular proliferation is a fundamental function of cells, enabling them to perform essential roles and support organ survival.^39^ During tumourigenesis, normal cells acquire malignant properties, including fast proliferation.^40^ PPARs, as transcription factors, play a role in cell proliferation and the dysregulation of apoptosis, consequently leading to tumourigenesis^41^; however, the isoform of PPARs contribute to tumour progression in different aspects.

PPAR‐α has been reported as a tumour promoter for the regulation of proliferation and cell death through lipid metabolic modulation in a breast cancer cell.^42^ Papi et al. reported the activation of PPAR‐α promoted multi‐signalling pathways, including nuclear receptor κB (NF‐κB)/interleukin‐6 (IL‐6) axis, and resulted in clonal expansion of breast cancer mammospheres.^43^ The dual role of PPAR‐γ in tumour survival is more complex than PPAR‐α. The anti‐proliferative and pro‐apoptotic properties of PPAR‐ γ have been widely reported in colon, oesophageal, breast, lung and prostate cancer.^44^, ^45^, ^46^ As a differentiation‐promoting factor, PPAR‐γ has been found as an antitumour target in breast cancer, although it is also associated with poor prognosis patients with Cox‐1 negative primary breast cancers.^47^, ^48^ Many studies have shown that the existing antidiabetic drug thiazolidinediones (TZDs), also a synthetic agonists of PPAR‐γ, exert a beneficial effect on breast cancer treatment in both a pre‐clinical mouse models and clinical trials.^49^ For instance, Khandekar et al. found PPAR‐γ ligands can induce cell death through accumulated DNA damage, which sensitize cancer cells to cytotoxic chemotherapy.^50^ However, in a Phase II clinical trial involving thyroid cancer, rosiglitazone, another member of the TZD family, did not show any relationship with the expression of PPAR‐γ.^51^ Similarly, PPAR‐γ play a dual role in prostate cancer and it may relate with complex factors including the research models and different stage of cancer progression. For example, PPAR‐γ was considered as a tumour suppressor due to its ability^52^ to inhibit tumour cell proliferation. However, it has been shown that a high level of PPAR‐γ expression is associated with late‐staged or high‐graded prostate cancer, suggesting its role in promoting prostate cancer.^53^ Furthermore, PPAR‐γ antagonists have also shown the anticancer effects in various epithelial cancer. The selective PPAR‐γ antagonists, T007, has been demonstrated the anticancer effect in breast cell lines MD‐231 and MCF‐7. It has also been shown that T007 is involved the apoptotic pathways in hepatocellular carcinoma cells in a certain concentration.^54^

## 3 PPARs INVOLVE TUMOUR DEVELOPMENT THROUGH METASTASIS

Metastasis is the process that cancer cells move from the primary site to form a new tumour in other parts of the body, which is usually associated with late‐stage cancer.^55^ In colon cancer, PPAR‐α promoted metastasis by inhibiting the expression of Cox‐2 and VEGF and TGF‐induced matrix metalloproteinase (MMP)‐9, both factors are highly implicated in metastasis promotion.^56^, ^57^ A study in melanoma revealed that the activation of PPAR‐γ is involved in the melanoma cell invasiveness through regulating thioredoxin‐interacting protein (TXNIP), integrin alpha‐v/beta‐3 and the tissue inhibitors of metalloproteinases (TIMP)‐2, all of which promoted the progression of metastasis in melanoma cancer (Figure 2B).^58^ It has been shown that both PPAR‐α and PPAR‐γ are widely involved in the late‐stage of cancer and promote metastasis. Especially, PPAR‐γ has been recognized as a therapeutic target for tumour angiogenesis and metastasis in multiple steps. For example, PPAR‐γ agonists could inhibit angiogenetic factors including FGF2 and VEGF, thus inducing endothelial cell apoptosis in several cancer cell lines.^52^

Many studies have shown that PPAR‐β/δ is associated with the progression of tumour metastasis across different cancer types. The activation of PPAR‐β/δ receptor has been widely shown to have a pro‐tumourigenic role in colon cancer progression, especially the APC mutation‐driven colorectal cancer progression^59^ (Figure 2C). A study showed that overexpression of PPAR‐β/δ is associated with poor prognosis colorectal cancer with potent of distant liver metastases.^60^ One possible explanation is that the PPAR‐β/δ receptor may be involved in cell proliferation. For example, the knock down of PPAR‐β/δ promotes the growth of colon cancer by reducing the differentiation and accelerating the proliferation in cell lines and xenograft mouse model.^61^, ^62^ Besides that, overexpression of PPAR‐β/δ can also increase migration and metastasis in breast cancer through elevated expression of antioxidant proteins and AKT‐mediated signalling, which are involved in the survival of breast cancer cells.^63^ In addition, PPAR‐β/δ receptor can stimulate the activation of β‐catenin and enhance invasiveness through PDGFR β, AKT1, EIF4G1 and CDK1 pathways.^64^, ^65^ Another explanation could be that the activation of PPAR‐β/δ receptor involves in the proinflammatory modulating and proangiogenic molecules across different cancer types.^59^

## 4 THE ROLE OF PPARs TME

The TME comprises nonmalignant cells including fibroblasts, endothelial cells, immune cells, as well as the acellular components like extracellular matrix, secreted chemokines, cytokines and cell metabolites. Over the past decades, the role of TME in tumour progression and the therapeutic resistance have become evident. Increasing appreciation gradually raised in the regulations of PPARs in TME cells which directly and indirectly exerted certain impacts on cancer progression.

### 4.1 PPARs in cancer stromal microenvironment

Under the paracrine influences of cancer cells, stromal cells such as CAFs and tumour‐associated endothelial cells can become the nutrients donor of tumour cells, providing fuels such as glutamine, L‐lactate, fatty acids and ketone bodies. PPARs govern many processes involved in the metabolic remodelling of stromal cells and further influent tumour cells.

#### 4.1.1 Fibroblast

As energy regulators, PPARs interact closely with metabolic regulator elements to reprogram the cell metabolism, and cell fate of fibroblasts. The PPARs‐regulated metabolic reprogramming in CAFs is correlated to tumour initiation, proliferation and progression through the epithelial–mesenchymal communication (Figure 4A). Clinically, the expression of PPAR‐γ is significantly upregulated in CAFs of cutaneous skin squamous cell carcinoma and colon adenocarcinoma.^66^ Avena et al. reported the overexpression of PPAR‐γ reprogrammed CAFs to the autophagic and glycolytic metabolism, which accelerates tumour growth in breast cancer xenograft mouse model when co‐implanted with transgenic PPAR‐γ‐high fibroblasts.^67^ PPAR‐β/δ in CAFs controls the redox homeostasis and affects tumourigenesis through stromal‐epithelial crosstalk. In skin tumour mouse model, Tan et al. showed, PPARβ/δ‐knockout fibroblasts remarkably increased the H_2_O_2_ production in the adjacent epidermis, subsequently triggering an RAF/MEK‐mediated NRF2 activation that elicits a strong antioxidant and cytoprotective response, which resulted in fewer and smaller skin tumours when comparing to wild‐type mice exposed to topical carcinogens.^68^ The expression of LRG1was upregulated by PPAR‐β/δ in fibroblasts. LRG1 interferes with TGF‐β1‐dependent redox homeostasis, resulting in oncogenic transformation in the surrounding epithelium.^69^, ^70^, ^71^

#### 4.1.2 Endothelium

Rapid tumour growth often induces hypoxic regions with poor oxygen perfusion and insufficient nutrients from the existing vasculature. This can be mitigated by secreted pro‐angiogenic factors which is promoted by PPAR‐β/δ, but inhibited PPAR‐α (Figure 4B). The pro‐angiogenic effects of PPAR‐β/δ activation have been revealed in previous studies. In PPAR‐β/δ knockout mouse model, the endothelial cells formed the immature microvessels in the tumours, leading to abnormal microvasculature and restricted blood flow into the tumours.^72^ The activation of PPAR‐β/δ in endothelial resulted in upregulated biosynthesis of VEGF, PDGFR and c‐KI, which accelerated endothelial cell proliferation and vascular formation.^73^ Beside conventional growth factors, other potential PPAR‐β/δ‐dependent angiogenic mediators include CDKN1C,^73^ IL‐8,^74^ CLIC4 and CRBP1.^75^ In contrast to PPAR‐β/δ, PPAR‐α is an anti‐angiogenic nuclear receptor. Activated PPAR‐α in stromal cells attenuated tumour angiogenesis and tumour xenograft growth by upregulating the expression of anti‐angiogenic factors, including thrombospondin‐1 and endostatin, which suppress endothelial cell proliferation and neovascularisation.^76^, ^77^, ^78^ PPAR‐γ was reported to maintain endothelium homeostasis through the interaction with key regulators of DNA repair signalling. Activated PPAR‐γ binds to DNA damage sensor MRE11‐RAD50‐NBS1 (MRN) and the E3 ubiquitin ligase UBR5, which promote ATM activation and DNA repair.^79^

### 4.2 PPARs in inflammation and cancer immune microenvironment

PPARs and their endogenous ligand lipids are closely related to the anti‐inflammation and immuno‐suppressive phenotype transformation of tumour‐infiltrated immune cells, which can be modulated either directly through regulating immunomodulatory gene expression or indirectly by altering lipid metabolism.

#### 4.2.1 Inflammation

**FIGURE 3:**
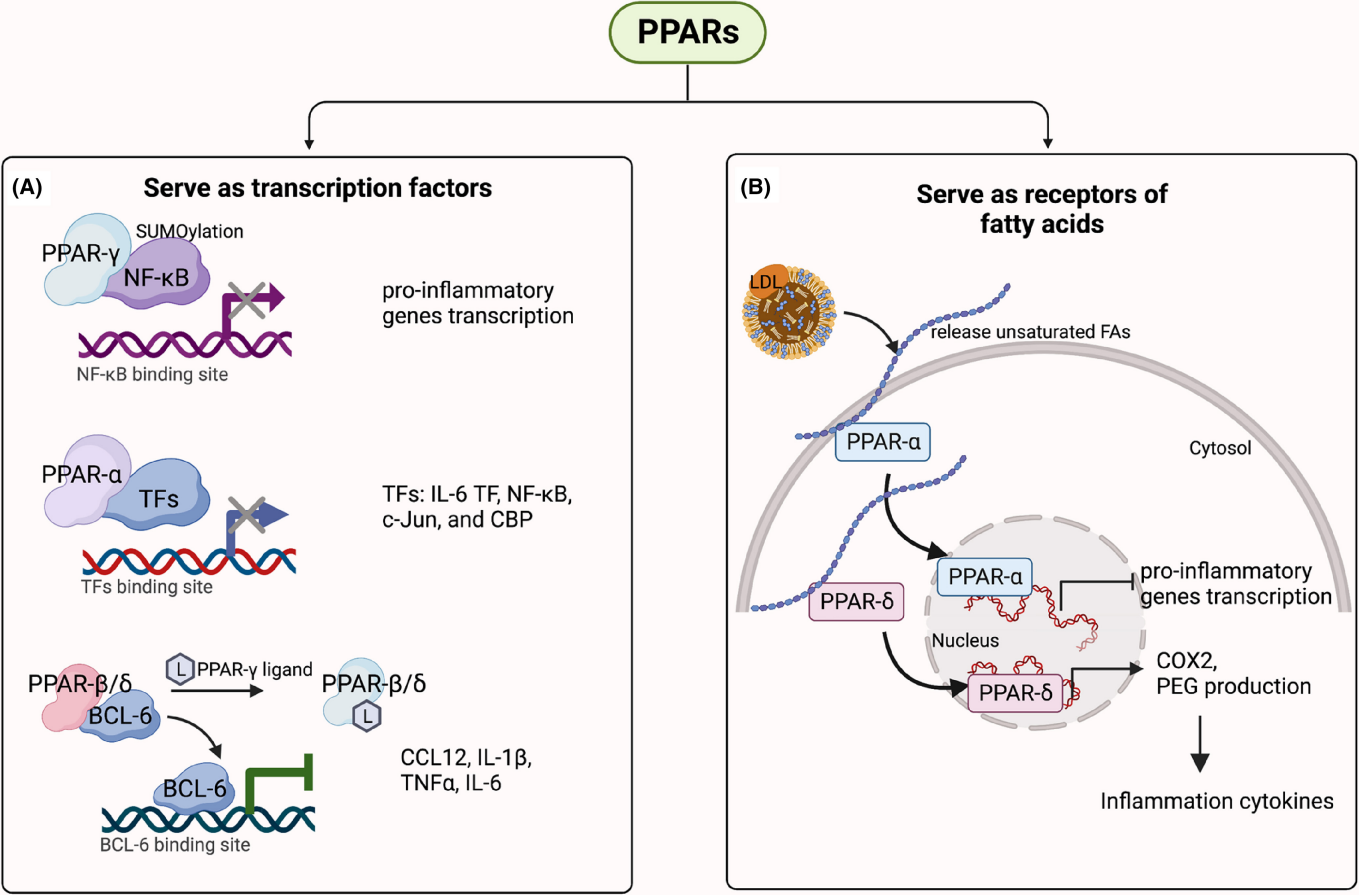
Role of peroxisome proliferator‐activated receptors (PPARs) in tumour inflammation. (A) PPARs serve as transcriptional regulators directly or indirectly repressing the expression of proinflammatory genes. Top, PPAR‐γ binds NF‐κB thus affect the transcription of the genes regulated by NF‐κB; middle, PPAR‐α binds transcription factors thus interfere the expression of IL‐6, the NF‐κB subunit p65, c‐Jun and c‐AMP response element‐binding protein‐binding protein (CBP); bottom, ligands activated PPAR‐β/δ releases BCL‐6 thus transrepress the expression of inflammatory genes. (B) PPARs are activated by lipid ligands further regulating the production of inflammatory factors. PPAR‐α is activated by FAs then translocate to nuclear repress the transcription of proinflammatory genes. In contrast, FAs activated PPAR‐δ induced the production of COX2 and PEG_2_ thus promoted the inflammation. Figure was generated with BioRender.

As transcription factors, activated PPARs bind to their recognition sequences and regulate the expression of genes involved in inflammation (Figure 3A). PPAR‐γ could stimulate the trans‐repression on proinflammatory genes like NF‐κB through a type of PTM called SUMOylation or through the conjugation with small ubiquitin‐like modifier (SUMO).^80^ Similarly, PPAR‐α could also downregulate inflammation by gene transrepression. It was found that PPARα directly bound key transcription factors of IL‐6, the NF‐κB subunit p65, c‐Jun and c‐AMP response element‐binding protein‐binding protein (CBP).^81^ To note, unlike the other two subtypes, PPAR‐β/δ ligand repress the inflammatory genes indirectly. Bartish et al showed activation of PPAR‐β/δ released BCL‐6 and trans‐repress the expression of inflammatory genes like CCL12, IL‐1β, TNFα, IL‐6.^82^

As receptors of fatty acid‐derived signals, PPARs are capable of transducing lipid‐mediated inflammatory signalling events^83^, ^84^ (Figure 3B). PPARs can directly modify the intracellular and extracellular lipid pool and alter the lipid microenvironment to initiate further inflammatory regulatory processes. It is noted that three subtypes of PPARs regulate lipid homeostasis in different ways. PPAR‐α provides energy from lipid catabolism during starvation, PPAR‐γ is activated in well‐fed state and initiate the synthesis of fatty acids, while PPAR‐β/δ regulates fatty acids level to ensure enough energy for muscles.^85^ Therefore, their regulations on inflammation are distinct. Many studies reported that activation of PPAR‐α and PPAR‐β/δ regulated the inflammatory process.^86^ For instance, low‐density lipoprotein (LDL) can release hydroxyoctadecadienoic acids (HODEs), known as PPAR‐α agonists, further reversing the proinflammatory responses of LDL.^87^ The proinflammation role of PPAR‐δ in tumours has been studied by various groups, especially in relation to lipid signalling like prostaglandin E_2_ (PGE_2_) induced inflammation process.^88^ Activation of PPAR‐δ in colon cancer cell lines upregulated COX‐2 expression and PGE_2_ production, subsequently increasing macrophage production of proinflammatory cytokines including CXCL1, CXCL2, CXCL4 and IL‐1β.^89^

#### 4.2.2 Cancer immune microenvironment

##### Myeloid

**FIGURE 4:**
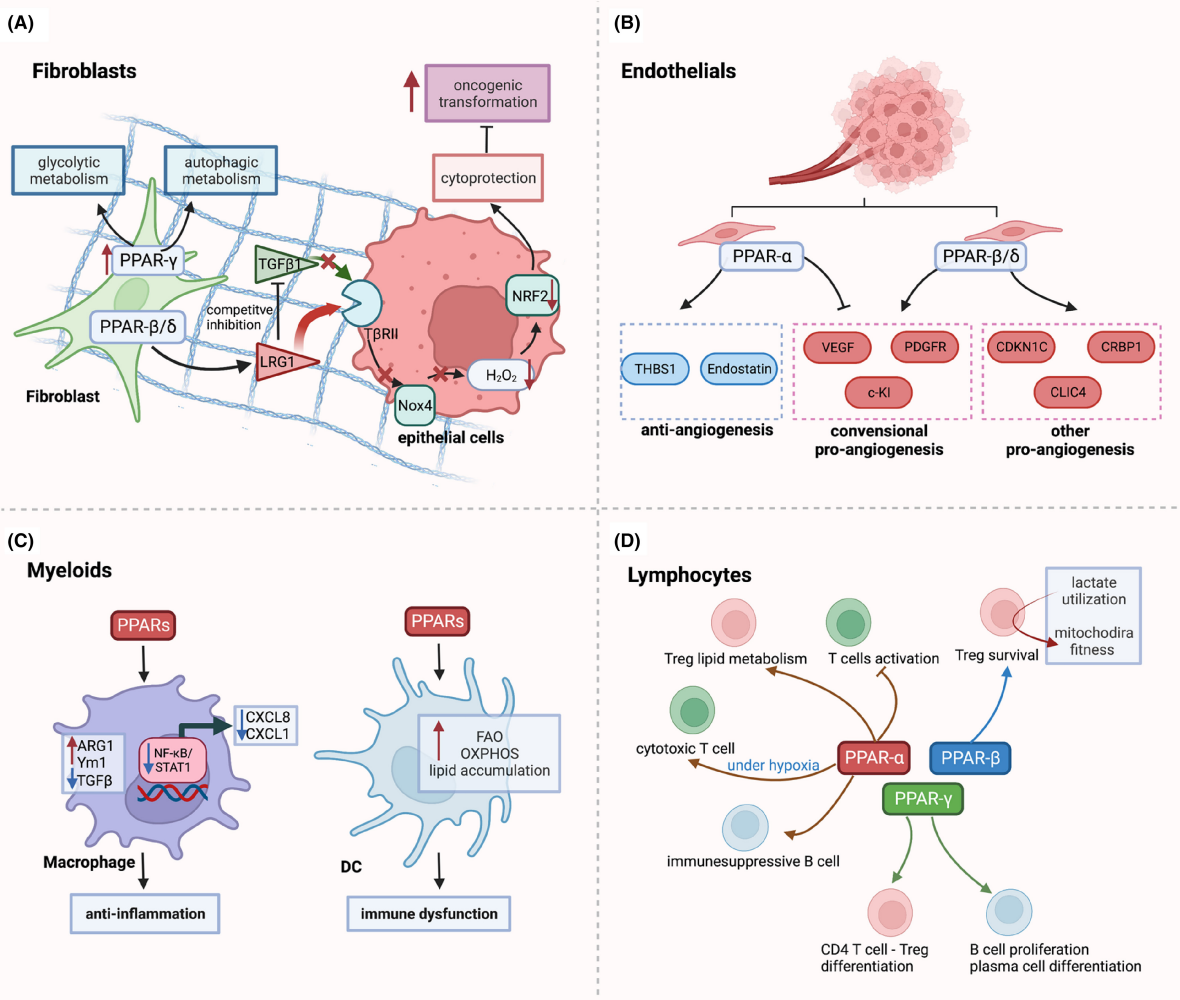
Role of peroxisome proliferator‐activated receptors (PPARs) in tumour microenvironment. (A) PPARs reprogram the metabolism of fibroblasts. overexpression of PPAR‐γ reprogrammed cancer‐associated fibroblasts to the autophagic and glycolytic metabolism, which accelerates tumour growth. PPAR‐β/δ activate LRG1 which interferes with TGF‐β1‐dependent redox homeostasis, resulting in oncogenic transformation in the surrounding epithelium. (B) In the endothelial cells, PPAR‐α exhibits an anti‐angiogenic effect by promoting anti‐angiogensis genes and inhibiting pro‐angiogenesis factors, while PPAR‐β/δ is more pro‐angiogenic, promoting endothelial proliferation and vascular maturation. (C) PPARs prime anti‐inflammatory M2‐like macrophage polarisation which causes anti‐inflammatory response; PPARs regulate DCs lipid metabolism, resulting in immune dysfunction. (D) PPARs involve in activation, differentiation, metabolic reprogramming of T cell and B cell. Figure was generated with BioRender.

Activation of PPARs in macrophages favours an anti‐inflammatory tumour‐associated macrophage (TAM) phenotype (Figure 4C).^90^ PPARs regulated the phenotypic changes of TAMs by gene transcriptomic modulation and enhanced fatty acid oxidation. In macrophages, PPAR‐α agonist and PPAR‐γ agonist‐induced M2 macrophage transformation by enhancing the expression of ARG1, Ym1 mannose receptor, TGF‐β and increasing phagocytic capacity.^91^ In primary human monocyte‐derived macrophages, PPAR‐δ ligands were reported to repress inflammation‐associated NF‐κB and signal transducer and activator of transcription 1 (STAT1)‐targeted genes, including CXCL8 and CXCL1, yielding the M2‐like macrophage phenotype.^92^ Recent studies have shown PPAR‐γ and PPAR‐δ primes M2 macrophage transformation by improving FAs metabolism and insulin sensitivity.^93^, ^94^ High concentrations of linoleic acid, arachidonic acid and lipid droplets in the TME can activate PPAR‐δ and polarize the pro‐tumoural TAMs in breast cancer and ovarian cancer.^95^, ^96^ Tumour‐infiltrating DCs (TIDCs) are critical in orchestrating antitumour immunity. DCs are more prone to adapt OXPHOS and FAO regulated by PPARs. Abnormal lipid accumulation is one of the emerging features of immune dysfunction of TIDCs. Yin et al. report that multitypes of tumour cells can activate PPAR‐α in TIDCs by secreting FA‐containing exosomes, resulting in lipid accumulation and FAO metabolic shift in TIDCs, ultimately leading to DC immune dysfunction.^97^

##### T and B lymphocyte

PPARs have been shown to regulate the activation, proliferation and differentiations in T cell and B cell.^98^ Unlike the dominant expression of PPAR‐γ in myeloids, PPARα is mainly found to express in T and B lymphocytes. It was reported that ligand activation of lymphocyte PPAR‐α antagonized NF‐κB and cytokine production then inhibited T‐cell activation.^99^ In addition, Wang et al. revealed that CD36–PPAR‐β signal orchestrates metabolic adaptation to lactate utilisation, which sustains survival and functional fitness of intra‐tumoural Treg cells, promoting survival of intra‐tumoural.^100^ Furthermore, activation of PPAR‐α and PPAR‐γ induced CD4^+^CD25^−^ T cells differentiated to functional Tregs by TGF‐β.^101^ PPARα signalling was reported to preserve cytotoxic function of tumour infiltrating CD8^+^ T cells through FAs catabolism under hypoglycaemic and hypoxic TME.^102^ Compared to T cells, the role of PPARs in B cells is not well explored. PPAR‐γ activation enhanced B‐cell proliferation and significantly stimulated plasma cell differentiation as well as Ab production.^103^ In contrast, Wejksza et al. reported that in breast cancer, PPAR‐α could be activated by tumour lipid metabolites, inducing immunosuppressive regulatory B cells and finally leading to distant metastasis (Figure 4D).^104^

## 5 PPARs COMBINATIONAL THERAPY TO TREAT CANCER

**FIGURE 5:**
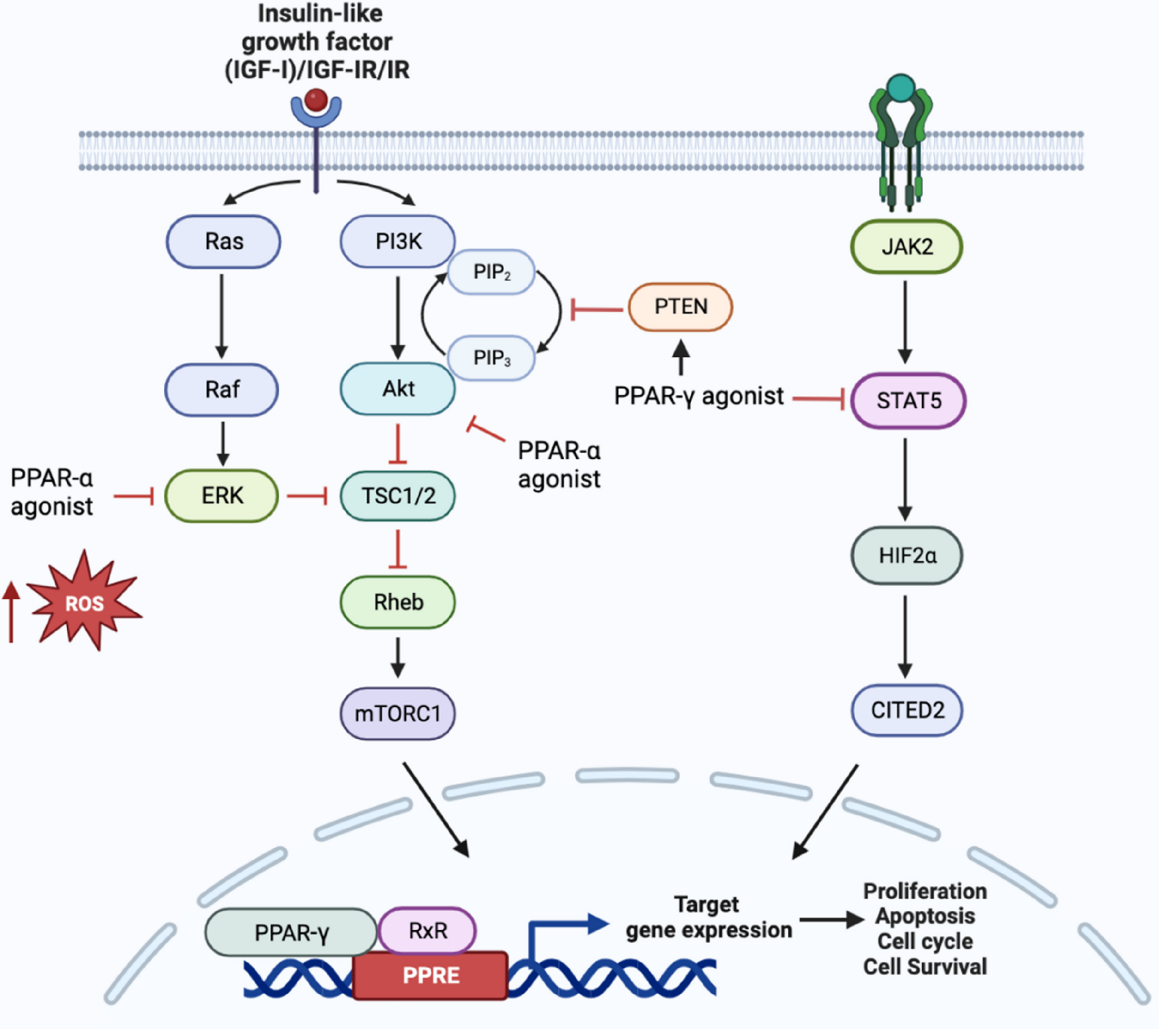
Schematic overview of the interaction of peroxisome proliferator‐activated receptor (PPAR) agonists with key signalling pathways. PPAR‐γ agonist enhance the expression of PTEN, which subsequently inhibits PI3K/AKT/mTORC1 pathway, resulting reduced tumour expansion and progression. Additionally, PPAR‐γ agonist can also downregulate the expression of STAT5 expression and its downstream targets, HIF2a and CITED2, promoting tumour quiescence. When combined with RXR agonist, PPAR‐γ agonist can potentiate their effects, leading to decreased cell proliferation and increased apoptosis by regulating the transcriptional activity of genes controlling these processes. On the other hand, PPAR‐α agonist, contribute to anticancer activity by elevating reactive oxygen species levels and inhibiting IGF‐I receptor signalling, thereby hindering tumour growth. Figure was generated with BioRender.

Despite the promising preclinical evidence, over the decade, monotherapy of PPARs agonists has not yielded exciting results for the treatment of advanced cancer, as many clinical trials showed mixed results. For example, troglitazone monotherapy showed little to no improvement on metastatic colon cancer and breast cancer,^105^, ^106^ and rosiglitazone had no or minimum effects on clinical Phase II studies for prostate^107^ and thyroid cancers.^108^ However, the use of PPAR agonists in combination with chemotherapy or other compounds has shown more promise. In this paper, we reviewed the potential of PPAR agonists to target signalling pathways and receptors for the treatment of cancer (Figure 5), as well as their potential for use in combination with immunotherapy and other cancer treatments to improve therapeutic efficacy. We also discussed how PPAR modulator could be combined with immunotherapy and other cancer treatments to boost the therapeutic efficacy (Table 1).

**TABLE 1:** Summary of peroxisome proliferator‐activated receptors (PPAR) combinational therapies.

Pathway	Subtypes	PPARs agonists	Combination	Cancer cells/function	Citation
PI3K/AKT/mTOR pathway	PPAR‐α	Clofibrate		Breast cancer cell	[37]
		Clofibric acid	Pioglitazone	Ovarian cancer	[35]
	PPAR‐γ	Balaglitazone	Celecoxib	Hepatocellular carcinoma expansion	[109, 110]
		Rosiglitazone	mTOR inhibitor rapamycin	Non‐small cell lung carcinoma cell	[111]
		N‐(9‐fluorenyl‐methyloxycarbonyl)‐l‐leucine (F‐L‐Leu)	Celecoxib	N1‐S1 cells, breast cancer, Huh7 cells	[110, 112]
		Pioglitazone	Arsenic trioxide	Leukaemia	[113]
		Pioglitazone	Trofosfamide, rofecoxib (a COX‐2 inhibitor)	Chemo‐refractory melanoma, soft tissue sarcoma, advanced vascular malignancies	[114, 115]
STAT5	PPAR‐γ	Glitazones	Imatinib mesylate	Chronic myeloid Leukaemia	[116, 117, 118, 119, 120]
Retinoid X receptor/retinoic acid receptor	PPAR‐γ	Ciglitazone, pioglitazone	Rexinoid 6‐OH‐11‐O‐hydroxyphenantrene (IIF)	Colon cancer cell lines	[121]
		Pioglitazone	IIF	Glioma cell lines, a murine glioma in vivo model	[122]
		Rosiglitazone, 15‐deoxy‐Delta 12,14‐prostaglandin J, triterpenoid 2‐cyano‐3,12‐dioxooleana‐1,9‐dien‐28‐oic acid	RXR agonist: LG100268	U937 and HL‐60; and lymphoid cells, including Su‐DHL, Sup‐M2, Ramos, Raji, Hodgkin's cell lines	[123]
		2‐cyano‐3,12‐dioxooleana‐1,9‐dien‐28‐oic acid	Bcl‐2 inhibitor HA14‐1	Bcl‐2‐overexpressing chronic lymphocytic leukaemia cells	[123]
Insulin‐like growth factor	PPAR‐α	Fenofibrate		Medulloblastoma cell line	[124]
		Fenofibrate		Glioma cells	[125]
Immunotherapy	PPAR‐α	Fenofibrate	PD‐1 blocker	Reprogramming the metabolism of effector T cells	[102]
	PPAR‐γ	Bezafibrate		Increase fatty acid oxidation and mitochondrial respiratory capacity in CD8^+^ T lymphocytes	[126]
		Ciglitazone	Lovastatin, phenylbutyrate	Trigger TNF‐α‐related apoptosis	[127]
Chemotherapy	PPAR‐γ	Pioglitazone	Cisplatinum	Orthotopic xenograft (PDOX) models of osteosarcoma	[128]
		Efatutazone	Paclitaxel	Advanced anaplastic thyroid carcinoma	[100, 129]
		Troglitazone	Tamoxifen	MCF‐7 cells	[130]
		Troglitazone	Lovastatin	DBTRG 05 MG (glioblastoma) and CL1‐0 (lung)	[131]
		Troglitazone	Aspirin	Lung cancer CL1‐0 and A549 cells	[132]
		Troglitazone	Radiation	Cervix cancer cells	[133]
		Troglitazone	Lovastatin	Human anaplastic thyroid cancer cell Line. Mouse xenograft model	[134]
		Troglitazone	TNF‐related apoptosis inducing ligand (TRAIL)	Breast cancer cell	[135]
		Troglitazone	Heregulin	Breast cancer cell	[136]
		Ciglitazone	TRAIL	Ca Ski cells containing HPV type 16	[137]
		Pioglitazone		Prevent radiation‐induced cognitive decline (RICD)	[138]

### 5.1 PPARs combinational therapy with targeted cancer essential signal pathways

#### 5.1.1 PI3K/AKT/mTOR pathway

The phosphatidylinositol 3‐kinase (PI3K)/AKT/mammalian target of rapamycin (mTOR) signalling pathway is essential in regulating cell proliferation, growth, metabolism, motility and cell size.^139^ The phosphatase and tensin homologue (PTEN) promoter is a natural inhibitor of PI3K/AKT pathway, and studies have shown that targeting PPARs can induce tumour regression by inducing PTEN expression. Two putative PPAR binding sites have been identified within PTEN promotor, suggesting that PPAR agonists may be able to modulate the PI3K/AKT/mTOR pathway and potentially have antitumour effects.^140^ A recent study suggests that Balaglitazone, a PPAR‐γ agonist, can reverse P‐glycoprotein‐mediated multidrug resistance by upregulating PTEN in a in leukaemia cell.^109^ The use of celecoxib, a COX‐2 inhibitor, has been shown to upregulate PTEN gene by activation of PPAR‐γ, leading to the inhibition of AKT and disruption of hepatocellular carcinoma expansion.^110^ Similarly, in a mouse model of breast cancer, the combination of celecoxib and the PPAR‐γ agonist N‐(9‐fluorenyl‐methyloxycarbonyl)‐l‐leucine (F‐L‐Leu) significantly delayed the tumour progression.^112^ Some clinical trials have been conducted to test the efficacy of PPAR‐γ as an adjuvant agent in treating patients with refractory or advanced cancer. For instance, a combinational of trofosfamide, rofecoxib (a COX‐2 inhibitor) and pioglitazone was found to have exerted encouraging results in patients with chemo‐refractory melanoma, soft tissue sarcoma, as well as advanced vascular malignancies, leading to stabilisation and remission.^114^, ^115^ Besides inducing PTEN expression, pioglitazone also intensified the tumour‐killing effect of arsenic trioxide (ATO) in leukaemia via the suppression of PI3K/AKT pathway,^113^ and mTOR inhibitor rapamycin has been shown to enhance the effects of rosiglitazone in inhibiting non‐small cell lung carcinoma (NSCLC) cell proliferation in vivo.^111^

In addition to PPAR‐γ, activation of PPAR‐α by its agonist clofibrate has also been shown to downregulate the inflammatory activity of COX‐2 and 5‐LO, and inhibits cell cycle‐related kinases and breast cancer cell survival.^37^ Another study in ovarian cancer showed that the combination of clofibric acid and pioglitazone significantly decreased the expression of COX‐2 and VEGF, leading to reduced tumour angiogenesis, tumour growth and induction of apoptosis through the inhibition of AP‐1.^35^

#### 5.1.2 STAT5

STAT5 proteins are recognized as major drivers in the development and/or maintenance of chronic myeloid leukaemia (CML).^116^ The development of tyrosine kinase inhibitors such as imatinib mesylate has revolutionized the treatment of CML; however, some patients do not respond well to this treatment due to high levels of STAT5 expression. Activation of PPAR‐γ by glitazones can re‐sensitize imatinib‐resistant CML to treatment by downregulating STAT5 expression and its downstream targets HIF2a and CITED2, two key guardians involved with quiescence and stemness of CML leukaemia stem cells. In a small trial, when pioglitazone was given temporarily to CML patients in chronic residual disease despite continuous treatment with imatinib, all of them achieved sustained complete molecular response even after withdrawal of the drug.^117^, ^118^, ^119^ The initial clinical Phase 1/2 trial investigating the combination of pioglitazone and imatinib in the treatment of CML patients was proven to be feasible and safe, although the efficacy of this therapy is being evaluated^120^ (Clinicaltrials.gov: NCT02852486).

#### 5.1.3 Retinoid X receptor/retinoic acid receptor

Many studies have shown that combination therapy using retinoid X receptor (RXR) agonist and PPAR‐γ agonist holds promise as novel therapy against cancers. In a study on colon cancer cell lines, the RXR agonist rexinoid 6‐OH‐11‐O‐hydroxyphenantrene (IIF) potentiated the antitumoural properties of PPAR‐γ agonist ciglitazone and pioglitazone in inhibiting cell growth and inducing apoptosis.^121^ The combination of IIF and pioglitazone also markedly reduced proliferation and induced apoptosis in three glioma cell lines, and reduced tumour volume and proliferation in a murine glioma in vivo model.^122^ PPAR‐γ ligation alone and in combination with either RXR agonist like LG100268 or a retinoic acid receptor agonist like all‐trans‐retinoic acid, has been shown to inhibit growth and enhance differentiating in myeloid cells (U937 and HL‐60) lymphoid cells (Su‐DHL, Sup‐M2, Ramos, Raji, Hodgkin's cell lines) and primary chronic lymphocytic leukaemia cells, by activating the transcriptional activity of target genes controlling apoptosis and differentiation in leukaemias.^123^

#### 5.1.4 Insulin‐like growth factor

It is now widely accepted that dysregulation of insulin‐like growth factor (IGF) signalling is involved in cancer development, progression and resistance. This signalling pathway involves the autocrine/paracrine production of IGFs (IGF‐I and IGF‐II) and overexpression of their cognate receptors (IGF‐I receptor, IGF‐insulin receptor (IR) and IR).^141^, ^142^, ^143^, ^144^ Fenofibrate, a PPAR‐α agonist, exerts an anticancer effect by accumulating reactive oxygen species (ROS) and inhibiting IGF‐I receptor signalling in glioma cells in vitro.^125^ It also attenuates IGF‐I‐induced phosphorylation of IRS‐1, AKT, ERKs and GSK3beta, and inhibits tumour growth in medulloblastoma cell lines.^124^ These findings suggest that the combination therapies of PPAR agonists may be effective in targeting IGF signalling in the treatment of cancer.

### 5.2 Combination of PPARs modulator with immunotherapy

In the recent decades, the cancer treatment era has been revolutionized by immunotherapy through modulating immune responses against tumour cells to overcome insufficient therapy such as radiotherapy and chemotherapy.^145^ However, there are still limitations to the use of immunotherapy. For example, while PD‐1 blockade can restore the function of effector T cells, these cells can still die from terminal differentiation and energy restriction in the tumour microenvironment. To address this, researchers have suggested combining drugs that modulate T cell metabolism with anti‐PD‐1 immunotherapy to enhance the antitumour activity of immunotherapy.^146^, ^147^, ^148^, ^149^ As a key regulator in tumour metabolism, PPAR agonists are good candidates for adjuvant to be used with immunotherapy to enhance their antitumour activity of active T cells, even in an immunosuppressive TME. Studies have shown that the PPAR‐α agonist fenofibrate can work synergistically with PD‐1 blockers in the immunotherapy of cancer by reprogramming the metabolism of effector T cells.^102^ Bezafibrate, an agonist of the PPAR‐γ coactivator 1α (PGC‐1α)/PPAR complex, has been shown to increase fatty acid oxidation and mitochondrial respiratory capacity in CD8^+^ T lymphocytes. This leads to an increase in mitochondrial oxidative phosphorylation and glycolysis, which can enhance antitumour immunity during PD‐1 blockade.^126^ Moreover, the co‐administration of ciglitazone, the first thiazolidinediones with drugs such as lovastatin and phenylbutyrate, which are not traditionally used as cancer medications, can trigger TNF‐α‐related apoptosis inducing‐ligand, enhance the effects of gamma‐radiation and lead to a decreased cancer cell viability,^127^ suggesting that these drug combinations could potentially be used as a treatment approach for cancer.

### 5.3 Other PPAR combinational therapies

PPAR agonists have been shown to have synergic effects when used in combination with chemotherapy, radiotherapy or even prevent side effects of these treatment regimens. Chemotherapy agents that induce the production of ROS have been shown to have higher efficacy when combined with a PPAR agonist. For example, in a patient‐derived orthotopic xenograft model of osteosarcoma, a combination of cisplatinum (CDDP)–pioglitazone (PIO) resulted in the greatest reduction in tumour volume and the most necrosis observed in histological sections.^128^ A recent Phase I clinical trial using efatutazone, a highly selective PPAR‐γ agonist,^129^ in combination with the microtubule inhibitor paclitaxel, demonstrated positive results in terms of safety, disease control and disease stability in patients with advanced anaplastic thyroid carcinoma (Clinicaltrials.gov: NCT00603941).^150^ Troglitazone has also been shown to have synergistic effects when used in combination with the lovastatin, nonsteroidal anti‐inflammatory drugs such as aspirin, the oestrogen modulator tamoxifen and x‐ray therapy in the treatment of thyroid, glioblastoma, lung, breast and cervix cancers.^130^, ^131^, ^132^, ^133^, ^134^ The combined administration of cell signalling molecules, such as TNF‐related apoptosis‐inducing ligand and troglitazone achieved promising apoptotic results synergistically in ovarian and breast cancer cell lines that are resistant to conventional therapies.^135^, ^136^, ^137^ Furthermore, the PPAR agonist pioglitazone has also been studied for its ability to prevent radiation‐induced cognitive decline in non‐diabetic patients undergoing radiotherapy in a Phase I clinical trial (Clinicaltrials.gov: NCT01151670),^138^ suggesting huge potentials of PPAR agonist as an adjuvant when combined with other therapies for improving the effectiveness and reducing the side effects of cancer treatments.

## 6 CONCLUSION AND FUTURE PERSPECTIVE

In this paper, we review the basic understanding of PPARs and recent research on their roles in tumourigenesis and microenvironment. Recent development of cancer therapies such as chemo, radio and immunotherapy have significantly improved cure rates for many patients. Nevertheless, for some patients who are less responsive to these established therapies, the clinical response rate remains unsatisfactory and this is where the potential of PPARs modulators becomes particularly relevant.

It is worth noting that monotherapies of PPARs agonist have yield a mixed results and are not used in the clinic. For instance, studies on patients with tenosynovial giant cell tumour treated with PPAR‐γ agonist zaltoprofen (UMIN‐CTR: UMIN000025901)^151^ or patients with metastatic colon cancer and breast cancer treated with TGZ^105^, ^106^, ^152^ have shown little to no improvement. The reasons for the failed response to PPAR modulators are complex, and one possible explanation is that many of the patients enrolled in these trials are heavily treated with many other chemo or biological therapies, making it difficult to evaluate the clinical effects of PPARs therapies.^105^ In addition, PPAR modulators may have more of a preventive than a therapeutic role in cancer. For example, a study suggests that PPAR‐ γ may play a role in the prevention of APC‐related colorectal carcinogenesis.^153^

Although PPAR modulators alone have shown limited efficacy, the co‐administration of PPAR agonists with chemotherapy or immunotherapy could open up new possibilities for increasing the effectiveness and accountability of cancer treatment. Here, we also illuminated how PPARs, when used as an adjuvant or in combination with other conventional cancer therapies, can exert antitumour effects.

Many PPAR agonists have strong safety profiles and have been widely used for a long time, making them convenient candidates for repurposing as cancer treatments. However, there are some limitations to their use as adjuvant in cancer treatment. Current studies have revealed conflicting results on the role of the different isoforms of PPARs in different types of tumours. For example, pioglitazone has been shown to potentially increase the risk of bladder cancer in patients with type II diabetes with a strong dosage effect.^154^, ^155^, ^156^, ^157^, ^158^ Therefore, more studies and clinical trials are needed to assess their efficacy and safety in different kinds of cancers. Additionally, PPARs agonists are often combined with many other therapeutics in cancer treatment,^159^ making it difficult to delineate the specific contribution of each component to the overall effect. To address this, it will be necessary to use biomarkers or more robust analysis methods to target the effects of PPAR agonists and determine whether they have additive or synergistic effects in combination therapy, which may also provide insights for future trial designs.^160^ Moreover, safety profiles of PPARs combinational therapy should be carefully examined before and during any trials, given the potential for increased adverse effects with combinational therapy.^161^

## AUTHOR CONTRIBUTIONS

**Yuqing Wang:** Conceptualization (lead); investigation (equal); writing – original draft (equal). **Feifei Lei:** Conceptualization (equal); investigation (supporting); writing – original draft (supporting). **Yiyun Lin:** Conceptualization (equal); investigation (equal); writing – original draft (equal); writing – review and editing (equal). **Yuru Han:** Conceptualization (equal); investigation (equal); writing – original draft (equal); writing – review and editing (equal). **Lei Yang:** Conceptualization (equal); investigation (equal); project administration (lead); writing – original draft (equal); writing – review and editing (equal). **Huabing Tan:** Project administration (lead); writing – review and editing (equal).

## CONFLICT OF INTEREST STATEMENT

The authors declare that there is no conflict of interest regarding the publication of this paper.

## Data Availability

The data that supports the findings of this study are available in material of this article.
